# The Treatment of Diabetes

**Published:** 1903-10-24

**Authors:** 


### Hospital Clinics and Medical Progress.

### THE TREATMENT OF DIABETES.

Dr. Hutchison[1] in a clinical lecture on this subject delivered at the London Hospital pointed out that the main object of treatment in diabetes is to get the urine, and therefore the blood, free from sugar. By getting rid of sugar from the blood a great deal is being done to cure the condition, because it appears that the presence of sugar in the blood still further impairs the power of dealing with carbohydrates. It is often found that by giving the patient's tissues a rest from sugar for some time, their power of assimilating it undergoes a considerable degree of increase. Again, many of the most disagreeable symptoms of diabetes are directly due to the sugar in the blood, for instance the intense thirst, eczema, and pruritus. Many of the complications of diabetes are also brought about by the presence of sugar in the blood, such as local suppurations, diabetic cataract and retinitis, perforating ulcer, neuritis, and so forth.

In any given case where glycosuria is found to be present it is necessary to determine whether this is a permanent condition or merely a transient phenomenon. Having determined this point, if it is a genuine case of diabetes, it is most important to test the urine for the presence of acid products of metabolism, chiefly $\beta$-oxybutyric acid and allied substances. This must be done at the outset, not only to discover whether or not the patient is in any immediate danger, but also for guidance in treatment. The way to test for $\beta$-oxybutyric and its allies is to add perchloride of iron to some of the urine, and if a deep port-wine red colour appears the presence of these substances is indicated. The next question that arises is the degree of severity of the diabetes, and the only way to estimate this is by noting the effect upon the urine of certain restrictions in the diet. For this purpose it is necessary to gradually reduce the amount of carbohydrate in the food and seeing if the sugar disappears from the urine.

It is an important practical question to decide whether all the carbohydrate should be withdrawn at once, or whether this should be done gradually. Dr. Hutchison's experience points to the advisability of the latter method, as he has known cases develop diabetic coma shortly after an abrupt withdrawal of carbohydrates from the dietary, and he considers, therefore, that it is better to make the reduction slowly, and particularly so if the perchloride of iron reaction be present. It is highly dangerous to make sudden changes in the diet when this reaction is present. The best procedure is to put the patient on an ordinary diet for three or four days and at the end of that time to make a careful estimation of the total excretion of sugar. The carbohydrates are then reduced in successive stages and the effect of each step is noted. Sugar is first withheld, then such things as puddings and potatoes, and finally bread. In some cases it may be necessary to forbid milk on account of the lactose it contains. It is clear that some substitute for these carbohydrates must be provided. For this purpose fat must be employed. Fat is the only true and safe substitute for carbohydrate in the diet of a diabetic. The reason for this is that fat is the only constituent of the food out of which sugar cannot be formed, and the main thing to do in dealing with a diabetic patient is to educate him in the eating and digestion of fat. This is not always an easy matter seeing that most diabetics do not digest fat readily. Amongst the fats most easily assimilated are cream, bacon, butter, and salad oil, and these should be given in large quantities. One of the greatest dietetic hardships is to be deprived of bread, but the introduction of casein preparations, which form the basis of all the modern diabetic breads, has done much to remedy this difficulty. Gluten bread, though often recommended, is open to the objections that it is not free from starch, and it is so light that a great deal of it must be eaten to get even a moderate amount of nourishment from it. Casein breads are not open to these objections, and the particular one that Dr. Hutchison recommends is "Casoid Meal Bread." It is fairly palatable, is nourishing, and when eaten with butter is an extremely useful food for diabetics. Milk can often be taken with impunity, lactose being less harmful than most carbohydrates. If the lactose is harmful, a lactose-free milk can be obtained.

Dr. Hutchison gives the following as a sample of a strict diet for a diabetic patient:—For breakfast, bacon or buttered eggs, or both, or cold ham; casoid meal bread with plenty of butter; coffee and milk sweetened with saccharine. In the course of the morning a glass of sugar-free milk and perhaps a casoid rusk. For lunch any animal food, *e.g.*, fish, or cold meat, salad, with plenty of oil, cheese, and fresh fruit stewed with saccharine. In the afternoon he may have tea with plenty of cream, but no sugar, and a slice or two of casoid bread and butter. For dinner, any clear soup, fish, meat, game, or other form of animal food, baked custard made with sugar-free milk, cheese, or savoury, and a little fresh fruit. Alcohol may be of use and is not often harmful, but malt liquors should be witheld because of the dextrine and maltose which they contain.

In some instances the sugar will disappear from the urine even while the patient is still taking a certain amount of carbohydrate. In any case the principle must be to try and keep the patient's blood free from sugar as long as possible. In other instances, in spite of the strictest diet, the glycosuria continues. These are the most troublesome cases of all. The sugar is formed from the patient's tissues or the proteid in his food, and in such instances proteid food is often more injurious than carbohydrate, since from proteid can be formed not only sugar but oxybutyric acid, and the other bodies to which diabetic coma is due. The thing to do here is to give as much fat as possible, and it may be as well to allow a moderate amount of carbohydrate food, simply because it is a good fat carrier. Most patients can go on almost indefinitely so long as the daily excretion of sugar does not rise above 50 grms. It is therefore well, if the glycosuria cannot be made to disappear altogether, to endeavour at any rate to keep the daily excretion of sugar below 50 grms. There are other points besides diet to be attended to. It is especially important to warn the diabetic patient against exposure to cold or muscular fatigue. With regard to drugs, Dr. Hutchison does not consider them of much value in diabetes.

Diabetic coma is produced by the neutralising of the alkali in the blood by oxybutyric and other acids, so that insufficient alkali is left to carry away the carbonic acid, the patient being practically poisoned by his own carbonic acid. It is therefore necessary, whenever the perchloride of iron reaction is obtained with the urine, to administer alkalies in large doses. One or two ounces of bicarbonate of soda should be given every day; and if coma has already set in, intravenous injection of alkalies may be done with the faint hope of doing some good. With regard to the curability of diabetes, Dr. Hutchison is of opinion that if a patient's blood can be kept comparatively free from sugar for a sufficiently long period the disease may be cured.

[1] Edinburgh Medical Journal, October, 1903.

